# Dynamic imaging markers of incident microhemorrhages and superficial siderosis in the A4 study: A longitudinal person–interval analysis

**DOI:** 10.1002/alz.71792

**Published:** 2026-08-27

**Authors:** Callum Hill, Huw Morgan, Sofia Michopoulou, Mahesan Niranjan, Christopher M. Kipps

**Affiliations:** ^1^ Clinical and Experimental Sciences Faculty of Medicine University of Southampton Southampton UK; ^2^ School of Electronics and Computer Science University of Southampton Southampton UK; ^3^ Department of Medical Physics University Hospital Southampton NHS Foundation Trust Southampton UK; ^4^ University Hospital Southampton NHS Foundation Trust Southampton UK

**Keywords:** A4 Study, Alzheimer's disease, amyloid‐related imaging abnormalities, anti‐amyloid therapy, ARIA‐H, longitudinal MRI, microhemorrhage, risk prediction, solanezumab, superficial siderosis

## Abstract

**INTRODUCTION:**

Incident microhemorrhages and superficial siderosis are hemorrhagic magnetic resonance imaging (MRI) abnormalities relevant to amyloid‐related imaging abnormalities with hemosiderin deposition (ARIA‐H) risk stratification, but evidence exploring their short‐term dynamic predictors remains limited.

**METHODS:**

We analyzed longitudinal MRI data from the A4 study, constructing a discrete person–interval dataset for modeling the short‐term risk of incident hemorrhagic MRI abnormalities. Models incorporated baseline covariates, alongside dynamic variables of recent microhemorrhage accumulation and current microhemorrhage burden. The outcome was defined as two or more new microhemorrhages or one or more new superficial siderosis between consecutive MRI scans.

**RESULTS:**

Among 1069 participants (3647 intervals), 171 hemorrhagic events occurred. Both current burden (time‐to‐event: odds ratio [OR] 1.37, 95% confidence interval [CI] 1.09–1.73; all‐event: OR 1.24, 95% CI: 1.04–1.49) and recent microhemorrhage accumulation were independently associated with an increased risk of hemorrhagic events (time‐to‐event: OR 1.83, 95% CI 1.01–3.30; all‐event: OR 1.43, 95% CI: 1.00–2.04).

**DISCUSSION:**

Temporal imaging variables may provide independent prognostic information beyond baseline risk, supporting a dynamic model of hemorrhagic risk in Alzheimer's disease.

**CLINICAL TRIAL REGISTRATION:**

Clinical trial of Solanezumab for older individuals who may be at risk for memory loss (A4) (NCT02008357).

## Highlights

- Person–interval modeling was applied to A4 study data.
- Current microhemorrhage burden predicted short‐term hemorrhagic risk.
- Recent microhemorrhage accumulation was associated with hemorrhagic risk.
- Systolic blood pressure and amyloid burden contributed prognostic information.

## 1 BACKGROUND

Cerebral microhemorrhages (MCHs) and superficial siderosis are hemorrhagic magnetic resonance imaging (MRI) abnormalities observed in Alzheimer's disease, cerebral amyloid angiopathy (CAA), aging, and in clinical trials of anti‐amyloid therapies.^1^, ^2^, ^3^ These abnormalities are most commonly detected on MRI, particularly on gradient echo (GRE)/T2*‐weighted sequences.^4^ Although their exact etiology remains unclear and varies by context, they have been linked to CAA, vascular fragility, and, in the setting of effective anti‐amyloid therapies, amyloid clearance across vessel walls.^2^, ^4^, ^5^, ^6^

In the context of anti‐amyloid therapeutics, incident MCHs and superficial siderosis are commonly classified as amyloid‐related imaging abnormalities with hemosiderin deposition, or ARIA‐H.^2^ Given the increasing use of anti‐amyloid therapies, ARIA‐H has become a clinically relevant safety concern.^7^, ^8^ While often asymptomatic, accumulation of hemorrhagic lesions can lead to treatment interruption or discontinuation, as well as increased MRI monitoring burden.^8^, ^9^ Identifying individuals at elevated risk of incident hemorrhagic MRI abnormalities therefore is important for balancing treatment risks and benefits.^9^, ^10^

Although ARIA‐H refers to the same radiographic components analyzed here, namely incident MCHs and superficial siderosis, the term is often interpreted as implying a treatment‐emergent imaging abnormality occurring in the context of amyloid‐targeting therapy.^2^, ^11^, ^12^ Similar hemorrhagic MRI abnormalities may also arise as part of the natural history of Alzheimer's disease, CAA, or aging.^12^, ^13^ In the present analysis of the A4 study, we therefore refer primarily to incident hemorrhagic MRI abnormalities, defined as incident MCHs or superficial siderosis, while discussing their relevance to ARIA‐H risk stratification in anti‐amyloid treatment contexts.

Existing studies have identified baseline risk factors associated with the development of ARIA‐H, including baseline MCH burden, apolipoprotein E (APOE) ε4 carrier status, vascular risk factors, and amyloid burden.^14^, ^15^, ^16^ Baseline MCH count has been identified as a risk factor for ARIA‐H development, likely reflecting existing cumulative vascular injury.^14^ Genetic predisposition, and in particular APOE ε4 homozygosity, has been established as a strong predictor of hemorrhagic risk.^15^ Evidence linking vascular risk factors and amyloid burden with hemorrhagic MRI risk remains more limited in the current literature.

However, these predictors are typically assessed at baseline and used to estimate risk over the duration of a study, often focusing on outcomes at later time points. As a result, such predictors are typically treated as static risk factors, with hemorrhagic risk accordingly conceptualized as a fixed property, rather than a dynamic process with temporal fluctuations. Understanding and modeling the time‐varying nature of hemorrhagic risk in Alzheimer's disease therefore represents an underexplored opportunity with potential to improve clinical decision‐making and risk stratification.

We therefore explored whether a longitudinal, temporally explicit approach better characterizes the dynamics of hemorrhagic risk. Rather than treating hemorrhagic risk as fixed, modeling incident MCHs and superficial siderosis in discrete intervals between MRI scans allows emerging hemorrhagic lesions to be analyzed over time. This approach enables the simultaneous consideration of current accumulation (“how much damage exists”), and the lagged variable of recent accumulation (“how active the process is”), which can be conceptualized as reflecting the current pathological state and trajectory respectively. Distinguishing between these processes may allow for improved risk prediction and provide insight into the underlying processes and mechanisms driving incident MCHs and superficial siderosis.

In this study, we utilized data from the A4 trial, representing a large and well‐monitored cohort of cognitively unimpaired participants with elevated amyloid.^17^ In the A4 study, participants were randomized to treatment with solanezumab or placebo. As solanezumab had limited effects on cerebral amyloid burden, this cohort provides a robust sample for assessing the temporal dynamics of incident hemorrhagic MRI abnormalities in a setting with relatively limited influence from active amyloid clearance. Using a person–interval dataset constructed from consecutive MRI assessments, we evaluated the temporal evolution and the role of temporally dynamic predictors in the short‐term prediction of hemorrhagic risk. We specifically evaluated whether recent MCH accumulation independently predicts short‐term hemorrhagic lesion risk beyond baseline risk factors and current lesion burden.

## 2 METHODS

### 2.1 Study design

We conducted a secondary data analysis of the A4 study, which enrolled cognitively unimpaired individuals with elevated amyloid. We conducted a longitudinal analysis of MRI data collected within the study.

Inclusion criteria for the analysis were sufficient data to establish at least one longitudinal value for change in MCH count. As the primary analyses included prior‐interval MCH change as a lagged predictor, the regression models required at least three MRI assessments for inclusion (*N* = 1069 participants). The dataset was segmented into intervals, with the start and end of each interval delineated by MRI scans. MRI scans were interpreted externally by radiologists in the original A4 study.

**RESEARCH IN CONTEXT**

1. **Systematic review**: We searched PubMed and reviewed existing literature on hemorrhagic lesion risk. Prior studies have identified baseline predictors including apolipoprotein E (APOE) ε4 status, microhemorrhage burden, vascular risk factors, and amyloid burden. However, these predictors are typically assessed at baseline, with limited evaluation of short‐term or time‐varying hemorrhagic risk.
2. **Interpretation**: In this longitudinal analysis of the A4 study, we found that recent microhemorrhage accumulation and current microhemorrhage burden were independent predictors of hemorrhagic events. Sensitivity analyses supported current microhemorrhage burden as a more robust predictor of hemorrhagic risk across severity thresholds than recent accumulation. These findings provide evidence for a dynamic model of hemorrhagic risk, incorporating both process activity (recent accumulation) and disease state (current burden).
3. **Future directions**: Future work should evaluate whether dynamic risk measures improve clinical risk stratification in populations treated with effective amyloid‐clearing therapies and determine whether short‐term hemorrhagic activity predicts longer‐term clinical outcomes.

### 2.2 Person–interval dataset construction

**FIGURE 1:**
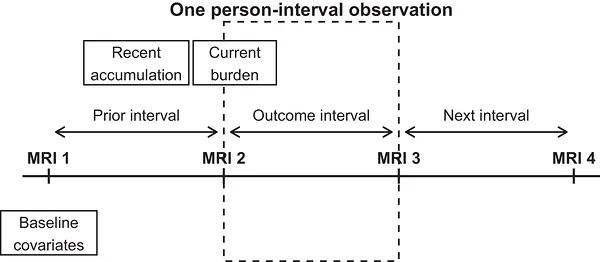
Person–interval dataset construction. Consecutive MRI scans defined discrete intervals. Current microhemorrhage burden was measured at the start of the outcome interval, while recent accumulation was defined as change in microhemorrhage count during the preceding interval. The same structure was repeated across eligible follow‐up intervals. MRI, magnetic resonance imaging.

MRI data were structured as a person–interval dataset. For each participant, MRI scans were ordered chronologically, and consecutive scans defined discrete time intervals. Each interval represented the period between two MRI assessments, during which incident hemorrhagic lesions could occur. Participants contributed multiple intervals where applicable. Demographic characteristics of the participants are displayed in Table 1. A schematic representation of the person–interval dataset construction is shown in Figure 1.

**TABLE 1:** Baseline characteristics of study participants.

Characteristic	Participants (*N* = 1140)
Age, years	71.9 (4.8)
Amyloid burden, Centiloids	66.0 (32.8)
WMH	5.0 (6.3)
Sex, *n* (%)	
Female	679 (59.6%)
Male	461 (40.4%)
Race, *n* (%)	
White	1074 (94.2%)
Black	27 (2.4%)
Asian	23 (2.0%)
Other	16 (1.4%)
APOE ε4 status, *n* (%)	
Non‐carrier	471 (41.3%)
Heterozygote	575 (50.4%)
Homozygote	94 (8.2%)
Systolic blood pressure, mmHg	136.6 (16.1)
Baseline microhemorrhage count, *n* (%)	
0	884 (77.5%)
1	181 (15.9%)
2 or more	75 (6.6%)
Baseline superficial siderosis, *n* (%)	
0	1125 (98.7%)
1 or more	15 (1.3%)
Treatment assignment, *n* (%)	
Solanezumab	558 (48.9%)
Placebo	582 (51.1%)

*Note*: Data are mean (SD) or *n* (%). Table includes participants in the person‐level analytic cohort used to construct the interval dataset.

Abbreviations: APOE, apolipoprotein E; WMH, white matter hyperintensity

For time‐to‐event analyses, a dataset was constructed in which participants contributed intervals up to and including the first qualifying event. A separate dataset including all intervals was retained for repeated‐interval analyses across the full follow‐up period.

### 2.3 Outcome

The study outcome was incident hemorrhagic MRI abnormality in any interval, defined as two or more new MCHs from the previous interval or any new superficial siderosis from the previous interval. As such, this represents a binary indicator of new hemorrhagic progression between consecutive scans. This threshold was chosen to represent a clinically meaningful increase in hemorrhagic activity.

### 2.4 Exposure and covariates

We explored both dynamic time‐dependent predictors as well as baseline covariates in modeling. Dynamic covariates included change in prior‐interval MCH count, as well as MCH count at the start of each interval.

Recent MCH accumulation was defined as the change in MCH count during the immediately preceding MRI interval. Current MCH burden was defined as the MCH count at the start of the interval.

Baseline covariates were selected based on biological plausibility and existing literature, and included treatment with solanezumab, APOE ε4 carrier state, age, systolic blood pressure, baseline amyloid, and white matter hyperintensity (WMH) tertile. Systolic blood pressure and amyloid Centiloid were scaled per 10 mmHg and per 10 Centiloids, respectively.

### 2.5 Statistical analysis

Discrete time‐interval logistic regression was used to model the probability of interval hemorrhagic progression. To account for within‐subject correlation arising from repeated intervals from the same participant, cluster‐robust standard errors were calculated.

A series of nested models were specified to investigate the relative contributions of dynamic variables to model fit in predicting hemorrhagic MRI abnormalities. Model 1 included only baseline covariates. Model 2 included MCH burden at the start of each interval to account for current lesion burden. Model 3 additionally included change in MCH burden in the previous interval. Model 4 included baseline covariates and prior‐interval MCH change without adjustment for current burden, allowing assessment of the total effect of recent accumulation independent of baseline factors. Model fit was compared using Akaike information criterion (AIC) and Bayesian information criterion (BIC), with lower values indicating better relative fit among models fitted to the same analytic sample.^18^, ^19^

As recent MCH accumulation was defined using the immediately preceding interval, dynamic models were restricted to intervals in which this prior change was available. To account for any time‐dependence of incident hemorrhagic lesions, models included MRI interval as a categorical variable. For valid model comparison, nested models were tested on the same analytic sample.

### 2.6 Temporal pattern of incident hemorrhagic MRI abnormalities

We investigated the temporal dynamics of hemorrhagic risk, and whether the risk of developing hemorrhagic lesions increases over time across strata defined by subject‐level risk factors. The time‐to‐event dataset was analyzed, with 1140 participants prior to the exclusion of missing prior‐interval MCH change. We limited the analysis period to include only the first five intervals, according to the MRI scans mandated by the study protocol.^17^

The cohort was stratified into groups based on subject‐level risk factors including age at baseline, treatment arm, APOE ε4 status, and WMH. We analyzed the interval hazard for each stratified group. Cumulative incidence values were derived by estimating cumulative hazard from interval‐specific Nelson–Aalen increments.^20^, ^21^

### 2.7 Sensitivity analysis

Sensitivity analyses were conducted to assess the robustness of the primary outcome definition. Models were repeated using a broader endpoint of one or more new MCH or one or more new superficial siderosis, and a stricter endpoint of four or more new MCHs or one or more new superficial siderosis. The same discrete‐time interval logistic regression framework was applied in both the time‐to‐event and all‐event datasets, adjusting for baseline covariates, MRI interval, current MCH burden, and prior‐interval change in MCH count, with cluster‐robust standard errors to account for repeated intervals within participants. Sensitivity analyses focused on whether the direction and magnitude of associations for current burden and recent accumulation were consistent across outcome thresholds.

## 3 RESULTS

### 3.1 Study population and dataset construction

**FIGURE 2:**
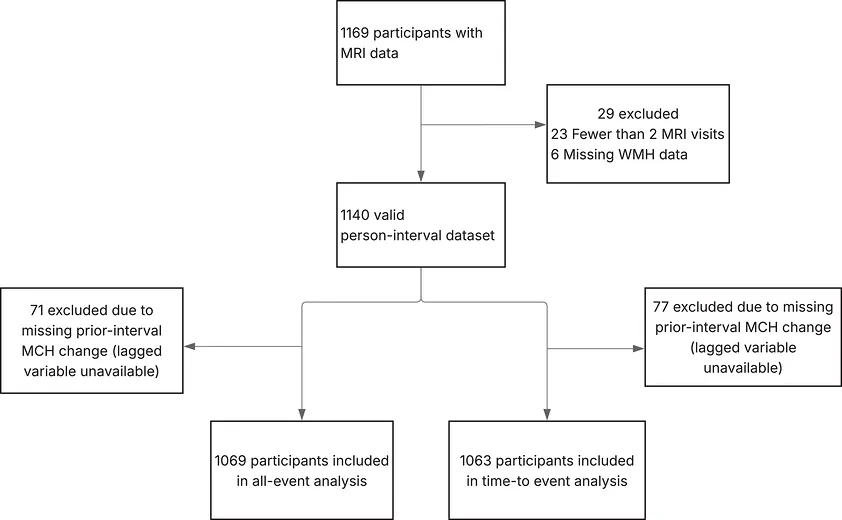
Study analytic populations. Participants with MRI data were used to construct a person–interval dataset after excluding those with missing data. Two analytic cohorts were defined: an all‐interval dataset and a time‐to‐event dataset. Participants were further excluded due to insufficient interval data to form the lagged prior‐interval MCH change predictor. MCH, microhemorrhage; MRI, magnetic resonance imaging.

Of the 1169 participants with MRI data, 1140 contributed to the interval dataset after excluding participants with insufficient MRI data or missing baseline covariates (Figure 2). In order for the lagged variable of prior‐interval MCH change to be defined, a minimum of two intervals with at least three MRI scans were required. The final datasets comprised 1069 participants in the all‐event analysis and 1063 participants in the time‐to‐event analysis.

A total of 171 hemorrhagic events occurred in the all‐event analysis from a total of 3647 intervals and 1069 participants included; 117 events occurred in the time‐to‐event analysis from a total of 3486 intervals and 1063 participants.

### 3.2 Distribution of hemorrhagic MRI events across intervals

Table 2 shows the interval‐risk of hemorrhagic events across successive intervals. Across successive intervals, the number of contributing participants declined from 1139 in interval 1 to 735 in interval 5. Interval hemorrhagic risk increased from 0.4% (95% CI: 0.1–1.0) to 6.8% (95% CI: 5.1–8.9) between interval 1 and interval 5. Correspondingly, person‐time incidence rates showed a similar pattern, rising from 1.38 per 100 person‐years (95% CI 0.45–3.23) in interval 1 to 8.70 per 100 person‐years (95% CI 6.46–11.47) in interval 5. These descriptive findings suggest an increased risk of hemorrhagic MRI abnormalities over time in the cohort studied.

**TABLE 2:** Interval‐specific incidence of hemorrhagic events across MRI intervals.

MRI interval	Participants contributing, *n*	Person–intervals, *n*	Mean interval start, days	Mean interval end, days	Mean interval length, days	Median interval length, days (IQR)	ARIA‐H events, *n*	Interval risk, % (95% CI)	Person‐years	Incidence rate per 100 PY (95% CI)
1	1139	1139	−29.5 (20.3)	86.3 (24.6)	115.8 (32.9)	111.0 (102.0–125.0)	5	0.4 (0.1–1.0)	361.1	1.38 (0.45–3.23)
2	1069	1069	86.2 (24.5)	543.2 (136.4)	457.0 (129.1)	501.0 (486.0–509.0)	31	2.9 (2.0–4.1)	1337.6	2.32 (1.57–3.29)
3	979	979	548.7 (130.9)	1065.4 (289.0)	516.7 (213.2)	586.0 (430.5–602.0)	43	4.4 (3.2–5.9)	1385.0	3.10 (2.25–4.18)
4	868	868	1059.9 (283.7)	1563.6 (337.5)	503.7 (156.5)	514.5 (478.0–587.0)	48	5.5 (4.1–7.3)	1197.1	4.01 (2.96–5.32)
5	735	735	1544.1 (337.0)	1829.8 (267.5)	285.7 (170.0)	198.0 (173.0–447.5)	50	6.8 (5.1–8.9)	574.9	8.70 (6.46–11.47)

*Note*: Data are mean (SD), median (IQR), *n*, or incidence estimates as indicated. Interval risk is the proportion of person–intervals with hemorrhagic events, with exact binomial 95% confidence intervals. Incidence rates are shown per 100 person‐years, with Poisson 95% confidence intervals.

Abbreviations: ARIA‐H, amyloid‐related imaging abnormalities with hemosiderin deposition; CI, confidence interval; IQR, interquartile range; MRI, magnetic resonance imaging; PY = person‐years.

**FIGURE 3 alz71792-fig-0003:**
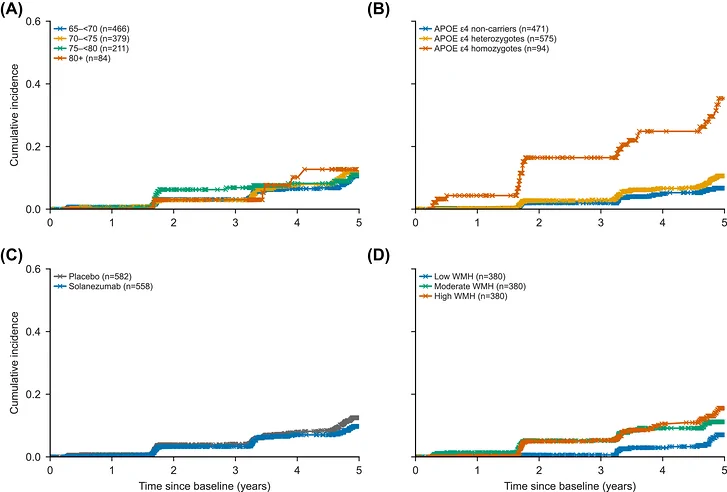
Cumulative incidence of hemorrhagic events by baseline subgroup. Descriptive cumulative incidence curves are shown according to (A) age group, (B) APOE ε4 status, (C) treatment arm, and (D) WMH tertile. Cumulative incidence was derived from the Nelson‐Aalen estimator. APOE, apolipoprotein E; WMH, white matter hyperintensity.

Figure 3 shows the cumulative incidence of hemorrhagic MRI abnormalities according to stratified subgroup. Sample sizes varied across analytic subgroups. The clearest separation was observed by APOE ε4 status, with homozygotes demonstrating a higher cumulative incidence throughout the study period. WMH tertile revealed some separation, with low WMH burden associated with a lower hemorrhagic risk. Cumulative incidence curves by treatment arm were broadly similar. Age‐stratified analysis revealed an increased risk over time in all subgroups, although there was no clear trend towards an increasing risk with age.

Descriptive cumulative incidence curves increased across all subgroups over the follow‐up period. Although cumulative incidence differed according to subject‐level risk factors, particularly APOE ε4 status and WMH burden, upward trajectories were observed across strata, consistent with a time‐dependent pattern of hemorrhagic risk.

### 3.3 Associations with interval hemorrhagic MRI abnormalities

**FIGURE 4 alz71792-fig-0004:**
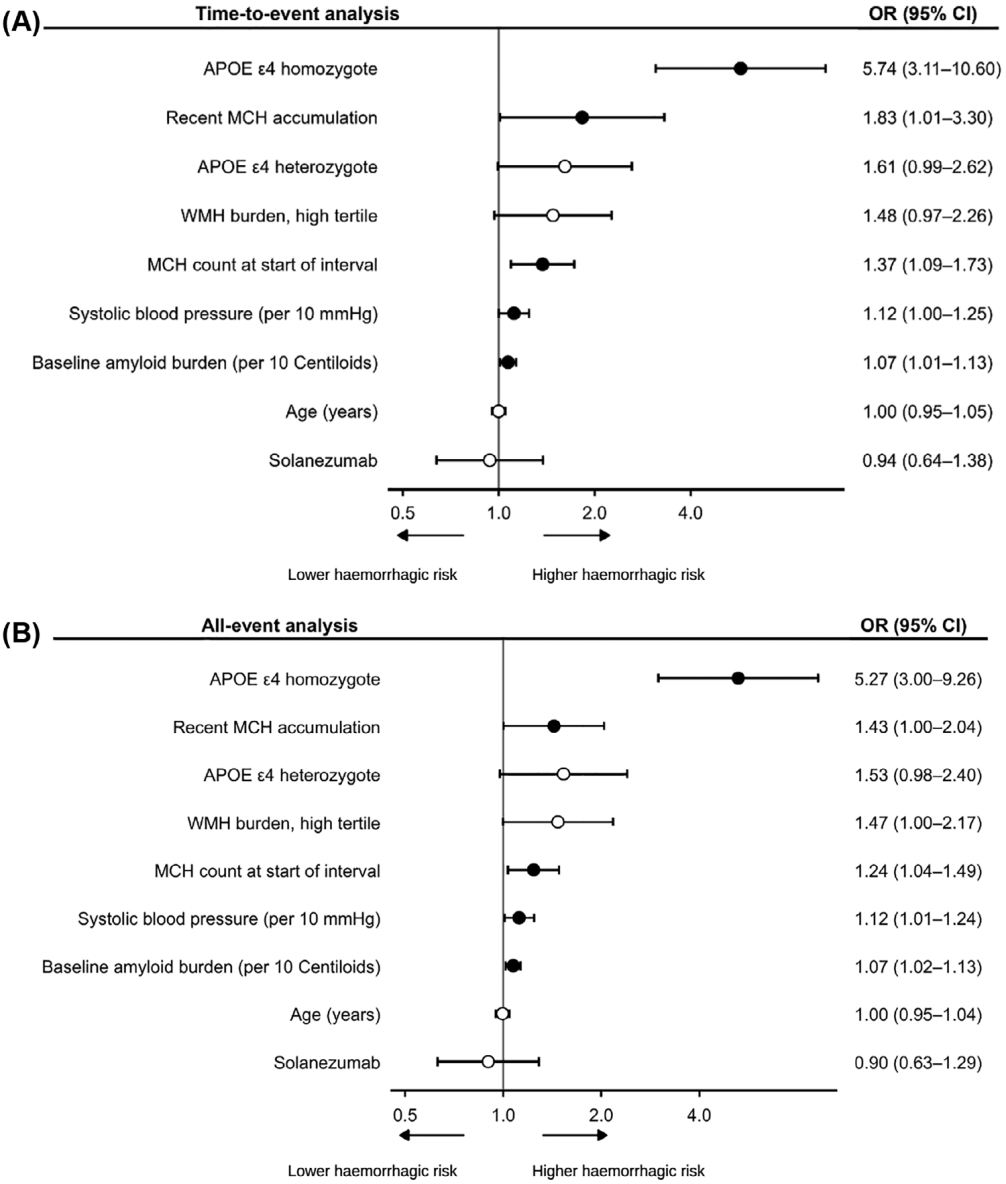
Forest plots show interval hemorrhagic events odds ratios for time‐to‐event analysis (A) and all‐event analysis (B). ORs and 95% CIs from clustered discrete‐time logistic regression models are shown. Points represent ORs and horizontal lines indicate 95% CIs; filled points denote *p* < 0.05. The *x*‐axis is on a logarithmic scale. CI, confidence interval; OR, odds ratio.

Figure 4 shows[Fig alz71792-fig-0003] odds ratios for each predictor used in the model, with cluster‐robust standard errors to account for repeated intervals for each participant. Both time‐to‐event associations and all‐interval associations were[Fig alz71792-fig-0004] modeled.

MCH burden at the start of each interval was associated with increased risk of hemorrhagic events (time‐to‐event: OR 1.37 95% CI 1.09–1.73; all‐event: OR 1.24 95% CI: 1.04–1.49). Recent MCH accumulation was associated with increased odds of hemorrhagic events after adjustment for baseline covariates, current MCH burden, and MRI interval (time‐to‐event: OR 1.83 95% CI 1.01–3.30; all‐event: OR 1.43 95% CI: 1.00–2.04).

Consequently, across the two analytic cohorts, both dynamic predictors were associated with a significant increase in the risk of hemorrhagic MRI abnormalities. These findings suggest that both recent hemorrhagic activity and current hemorrhagic burden convey prognostic information beyond baseline risk factors. The associations of both variables with hemorrhagic events when included in the same model suggest that they capture related but distinct aspects of subsequent hemorrhagic risk.

Across baseline covariates, APOE ε4 homozygosity was associated with the highest odds of hemorrhagic events in the analysis conducted. Higher baseline systolic blood pressure and amyloid burden were significantly associated with increased hemorrhagic risk in the study period (per 10 mmHg increase in systolic blood pressure: OR 1.12 95% CI 1.00–1.25 and OR 1.12 95% CI 1.01–1.24; per 10 Centiloid increase in amyloid burden: OR 1.07 95% CI 1.01–1.13 and OR 1.07, 95% CI 1.02–1.13 for the time‐to‐event and all‐interval analyses, respectively). Baseline WMH burden showed a positive association that did not reach statistical significance. ORs for treatment with solanezumab, age, and APOE ε4 heterozygosity were non‐significant.

In the sensitivity analysis conducted, current MCH burden remained consistent with increased risk of further hemorrhagic lesions. For the broader endpoint of one or more new MCH or one or more new superficial siderosis, current burden was associated with increased risk in both analyses (time‐to‐event: OR 1.57 95% CI 1.30–1.90; all‐event: OR 1.43 95% CI 1.25–1.64). Similar findings were observed for the stricter endpoint of four or more new MCH or one or more new superficial siderosis (time‐to‐event: OR 1.38 95% CI 1.12–1.70; all‐event: OR 1.21 95% CI 1.05–1.41).

Recent accumulation showed positive but non‐significant associations for both alternative endpoints (one or more endpoint: time‐to‐event OR 1.35 95% CI 0.89–2.04; all‐event OR 1.13, 95% CI 0.88–1.46; ≥4 endpoint: time‐to‐event OR 1.13 95% CI 0.46–2.80; all‐event OR 1.27 95% CI 0.95–1.70). Full results of the sensitivity analysis can be found in File S1.

### 3.4 Model performance for prediction of interval hemorrhagic events

The performance of four discrete‐time interval logistic regression models is shown in Table 3. Performance was assessed separately across the time‐to‐event and all‐event analysis. Discrimination was assessed using area under the receiver operating characteristic curve (AUC). Model fit was assessed using AIC and BIC.

**TABLE 3 alz71792-tbl-0003:** Comparison of discrete‐time logistic regression models for prediction of interval hemorrhagic events.

Model	Events	AIC	BIC	AUC (95% CI)
Time‐to‐event analysis (3486 intervals; 1063 participants)				
Model 1: baseline risk only	117	986.6	1054.3	0.70 (0.69–0.75)
Model 2: baseline risk + current burden	117	960.5	1034.4	0.74 (0.72–0.80)
Model 3: baseline risk + current burden + recent accumulation	117	958.0	1038.1	0.74 (0.71–0.78)
Model 4: baseline risk + recent accumulation	117	973.6	1047.5	0.72 (0.70–0.77)
All‐event analysis (3647 intervals; 1069 participants)				
Model 1: baseline risk only	171	1273.7	1341.9	0.74 (0.72–0.80)
Model 2: baseline risk + current burden	171	1153.9	1228.3	0.80 (0.76–0.85)
Model 3: baseline risk + current burden + recent accumulation	171	1145.2	1225.8	0.80 (0.77–0.84)
Model 4: baseline risk + recent accumulation	171	1160.7	1235.1	0.78 (0.75–0.82)

*Note*: Upper panel shows time‐to‐first‐event analysis; Lower panel shows all‐event analysis. Models were fitted separately with baseline covariates alone, followed by the introduction of temporal covariates in models 2–4. Model fit was assessed using AIC and BIC, and discrimination using AUC. Confidence intervals for AUC were derived from participant‐level bootstrapping.

Abbreviations: AIC, Akaike Information Criterion; AUC, area under the receiver operating characteristic curve; BIC, Bayesian Information Criterion.

Inclusion of current MCH burden was associated with a modest increase in model discrimination compared to baseline covariates alone. In the time‐to‐event analysis, AUC improved from 0.70 in Model 1 to 0.74 in Model 2; in the all‐event analysis, AUC improved from 0.74 in Model 1 to 0.80 in Model 2. Corresponding reductions in AIC and BIC indicated improved model fit across both analyses.

Further inclusion of recent MCH accumulation in addition to current burden resulted in minimal changes to model discrimination. In the time‐to‐event analysis, the addition of recent accumulation resulted in a small reduction in AIC (ΔAIC = 2.5), but no improvement in BIC. In the all‐event analysis, both AIC and BIC improved following incorporation of recent accumulation.

Model 4 included only baseline covariates and recent accumulation to ascertain the independent effect of recent accumulation. Model fit improved and discrimination showed a modest increase in comparison to the baseline risk model.

## 4 DISCUSSION

In this longitudinal[Table alz71792-tbl-0003] analysis of the A4 study, both recent MCH accumulation and current MCH burden were independently associated with an increased risk of incident MCHs and superficial siderosis. Our results support the conceptualization of hemorrhagic risk as a dynamic property, rather than as a fixed quantity observed prior to trial enrollment or treatment initiation. However, sensitivity analyses provided more robust support for current MCH burden as an interval‐level predictor, while the association with recent accumulation was less consistent across alternative outcome definitions.

Although related, current burden and recent accumulation appear to capture distinct but complementary aspects of hemorrhagic progression: current burden reflects disease state, whereas accumulation reflects recent trajectory and short‐term risk. Importantly, these associations were observed even after accounting for MRI interval, suggesting that recent accumulation was not merely a proxy for later follow‐up time.

The results of model fit metrics provide further support for the utility of dynamic variables. Inclusion of current burden produced the clearest improvement in performance, with lower AIC and BIC and modestly higher AUC in both the time‐to‐event and all‐event analyses. This is consistent with the interpretation that current MCH burden captures a component of hemorrhagic susceptibility.^10^ Recent accumulation showed mixed incremental value, with stronger support in the all‐event analysis than the time‐to‐event analysis.

Sensitivity analyses using both broader and stricter hemorrhagic event definitions support the interpretation that there is more robust evidence supporting the association of current MCH burden than recent MCH accumulation. Current MCH burden remained independently associated with hemorrhagic events across thresholds, whereas recent MCH accumulation showed directionally positive but non‐significant associations under alternative thresholds.

Sensitivity analyses were not conducted for MCH thresholds above four or more due to insufficient sample size under such thresholds, and subsequent model instability. The lower threshold of one or more new MCH or one or more new superficial siderosis increased event counts substantially, but may be more sensitive to stochastic variation in image reporting or acquisition. Conversely, the stricter threshold of four or more new MCHs or one or more new superficial siderosis was limited by smaller event counts and potential model instability.

Unsurprisingly, APOE ε4 homozygosity was the strongest predictor of hemorrhagic events among participants in our analysis. The effects of age, APOE ε4 heterozygosity, and solanezumab were non‐significant. Higher baseline WMH trended toward an increased risk, although did not reach significance in either analytic cohort. Amyloid burden and baseline systolic blood pressure showed modest positive associations with hemorrhagic lesion risk. These findings are biologically plausible given the association of amyloid burden with angiopathy, and the potential for chronic hypertension to increase hemodynamic stress on already fragile amyloid‐affected small vessels and promote endothelial and blood–brain barrier dysfunction.^4^, ^5^, ^22^, ^23^, ^24^, ^25^

Biologically, recent MCH accumulation may represent a period of heightened vascular instability, rather than chronic lesion accumulation at a constant rate. In the context of CAA, possible explanations for this include transient worsening of vessel‐wall fragility, blood–brain barrier and perivascular dysfunction, or local inflammatory activity, each of which could increase the likelihood of further short‐term leakage.^23^, ^26^, ^27^, ^28^ The observation of this pattern in the A4 study, in which amyloid clearance was limited and solanezumab was not associated with increased hemorrhagic risk, suggests that it may reflect an underlying disease‐related process rather than a purely treatment‐induced effect.^17^ However, it remains unclear whether the observed associations reflect cerebral amyloidosis as opposed to microvascular changes related to non‐amyloid small‐vessel disease.

Our results have implications for ARIA‐H risk‐stratification on a clinical level in the context of anti‐amyloid therapies, where clinical decisions depend on assessment of short‐term hemorrhagic risk.^9^, ^10^ Dynamic measures may have utility in complementing established baseline risk factors for predicting short‐term ARIA‐H risk by identifying periods of vascular vulnerability. If validated in clinical cohorts receiving effective amyloid‐clearing therapies, dynamic risk metrics may guide decision‐making surrounding treatment discontinuation or suspension, recommended frequency of MRI monitoring, and individual‐level risk counseling. The associations observed for amyloid burden and systolic blood pressure suggest a role for integrating these baseline risk factors in modeling ARIA‐H risk, as well as their use in clinical risk‐stratification. Although this study did not aim to develop a prediction model, and the results do not yet support direct clinical implementation, they suggest that dynamic risk metrics may have a role in future risk stratification and clinical decision making.

### 4.1 Comparison to existing literature

Longitudinal studies provide complementary evidence that cerebral MCHs can accumulate over time. In population‐based aging cohorts, incident cerebral microbleeds have been observed during follow‐up, with incidence associated with older age, baseline microbleed burden, vascular risk factors, and amyloid burden.^3^, ^13^, ^29^ Similar longitudinal progression has been reported in memory‐clinic and preclinical Alzheimer's disease cohorts, where incident microbleeds were associated with baseline microbleeds, amyloid burden, and concomitant cerebrovascular disease markers.^30^, ^31^ In CAA cohorts, hemorrhagic MRI markers, including lobar microbleeds and cortical superficial siderosis, also show longitudinal progression.^32^, ^33^

Prior studies have primarily focused on baseline risk factors for hemorrhagic MRI abnormalities, largely in the context of effective amyloid‐clearing therapeutics. In the existing literature, the primary analytic approaches include observation at the start and endpoint of studies, as well as time‐to‐event analysis.^14^, ^16^ Baseline MCH count, although not the primary focus of this study, has been established as a predictor of future hemorrhagic risk.^2^, ^14^ To our knowledge, current studies have largely focused on baseline predictors, and we did not identify any studies explicitly modeling current lesion burden and recent MCH accumulation as separate time‐varying predictors of short‐term hemorrhagic lesion risk.

Our findings are consistent with the existing literature on genetic risk factors, with APOE ε4, especially homozygosity, being a strong risk factor for hemorrhagic lesions.^2^, ^6^ The association between amyloid burden and hemorrhagic MRI abnormalities is theoretically plausible, although evidence supporting the association is variable.^4^, ^15^, ^16^ Hypertension has been suggested as a potential risk factor for ARIA more generally (including ARIA‐E) in the current literature, although evidence linking hypertension with ARIA‐H in the context of amyloid‐therapeutics is limited.^7^, ^12^, ^34^, ^35^

Although Shirzadi et al. (2025) also analyzed hemorrhagic MRI abnormalities in A4, their study related baseline factors to final hemorrhagic lesion status, whereas the present analysis modeled scan‐to‐scan risk. Shirzadi et al. reported an independent association between elevated WMH burden and hemorrhagic lesion emergence, whereas WMH showed only a positive but non‐significant association with interval risk in the present analysis, potentially reflecting differences in analytic design or inclusion criteria.^16^

### 4.2 Strengths and limitations

Strengths of this study include the robust dataset; the A4 study allows for analysis of a well‐monitored cohort of participants according to a pre‐defined protocol.^17^ Furthermore, the study implemented a standardized protocol for central reading of MRI scans, improving the validity of analyses conducted. Our discrete person–interval approach provides a coherent framework for modeling MRI‐detected events between scheduled scans. We also conducted both time‐to‐event and all‐event analyses and used cluster‐robust standard errors to account for repeated participant intervals.

However, several limitations should be acknowledged. It should be noted that the A4 study was conducted in a selected, predominantly white, pre‐symptomatic sample of participants with preclinical Alzheimer's disease.^17^ As such, our findings may not fully generalize to symptomatic individuals or routine clinical cohorts.

The potential effect of amyloid‐clearing therapeutics on our results remains unclear. Further research is needed to demonstrate whether our results translate to cohorts treated with contemporary anti‐amyloid therapies. Our outcome definition was chosen as a clinically meaningful benchmark for incident hemorrhagic MRI abnormality, although other endpoint thresholds could be justified, likely with differing results. We note that incident MCHs and superficial siderosis are measured between MRI scans, and differences in interval spacing may introduce uncertainty in our results. Even with central MRI reading, some variability exists in image reporting, with the additional variable of MRI magnetic field strength potentially affecting hemorrhagic lesion readings.^36^, ^37^ While independent associations and improvements in model fit were observed, improvements in model discrimination were modest throughout the analysis. Although the development of a validated prediction model is beyond the scope of this study, we acknowledge that external validation would provide evidence for the generalizability of our results. Finally, as a secondary observational analysis, this study cannot establish causal relationships between covariates and subsequent incident hemorrhagic MRI abnormalities.

## 5 CONCLUSIONS

Our results suggest that both recent MCH accumulation and current MCH burden are independently associated with subsequent incident hemorrhagic MRI abnormalities in the primary endpoint analysis. These findings support the conceptualization of hemorrhagic risk as a dynamic process in which current lesion burden, alongside recent lesion activity, may improve short‐term risk prediction. Given the recent introduction of amyloid‐clearing therapies into clinical practice, both improving the understanding of hemorrhagic risk in treated populations, and individual‐level temporal prediction of hemorrhagic risk may inform clinical decision‐making and monitoring strategies. If validated in suitable cohorts receiving anti‐amyloid therapies, our findings may inform a more individualized and dynamic approach to ARIA‐H risk assessment and monitoring.

## CONFLICT OF INTEREST STATEMENT

Christopher M. Kipps delivers industry‐sponsored trials, provides consultancy, and has accepted hospitality from Biogen, Eisai, Lilly, Novartis, and Roche. All other authors declare no conflicts of interest. Author disclosures are available in the Supporting Information.

## ETHICAL APPROVAL

This study was a secondary analysis conducted in accordance with the applicable data‐use agreement. Ethical approval was granted by the Faculty of Medicine Ethics Committee (ERGO:112166).

## CONSENT STATEMENT

This study was a secondary analysis of de‐identified data from the A4 study. Participant consent was obtained as part of the original A4 study procedures.

## Supporting information




**Supporting Information**: alz71792‐sup‐0001‐SuppMat.xlsx

**Supporting Information**: alz71792‐sup‐0002‐SuppMat.pdf

## Data Availability

The data that support the findings of this study are available to researchers following approval through the Alzheimer's Clinical Trial Consortium A4/LEARN data portal: https://www.a4studydata.org.
